# Controllable Synthesis of 2D Perovskite on Different Substrates and Its Application as Photodetector

**DOI:** 10.3390/nano8080591

**Published:** 2018-08-03

**Authors:** Yunzhou Xue, Jian Yuan, Jingying Liu, Shaojuan Li

**Affiliations:** 1 College of Chemistry and Environmental Engineering, Shenzhen University, Shenzhen 518000, China; xueyz@iccas.ac.cn; 2 Collaborative Innovation Center of Suzhou Nano Science and Technology, Jiangsu Key Laboratory for Carbon-Based Functional Materials and Devices, Institute of Functional Nano and Soft Materials (FUNSOM), Soochow University, Suzhou 215123, China; jianyuan_cq@126.com; 3 Department of Materials Science and Engineering, Monash University, Clayton, VIC 3800, Australia; jingying.liu@monash.edu

**Keywords:** 2D perovskite, controllable synthesis, flexible substrate, photodetector, photoelectric performance

## Abstract

Perovskites have recently attracted intense interests for optoelectronic devices application due to their excellent photovoltaic and photoelectric properties. The performance of perovskite-based devices highly depends on the perovskite material properties. However, the widely used spin-coating method can only prepare polycrystalline perovskite and physical vapor deposition (PVD) method requires a higher melting point (>350 °C) substrate due to the high growth temperature, which is not suitable for low melting point substrates, especially for flexible substrates. Here, we present the controlled synthesis of high quality two-dimensional (2D) perovskite platelets on random substrates, including SiO_2_/Si, Si, mica, glass and flexible polydimethylsiloxane (PDMS) substrates, and our method is applicable to any substrate as long as its melting point is higher than 100 °C. We found that the photoluminescence (PL) characteristics of perovskite depend strongly on the platelets thickness, namely, thicker perovskite platelet has higher PL wavelength and stronger intensity, and thinner perovskite exhibits opposite results. Moreover, photodetectors based on the as-produced perovskite platelets show excellent photoelectric performance with a high photoresponsivity of 8.3 A·W^−1^, a high on/off ratio of ~10^3^, and a small rise and decay time of 30 and 50 ms, respectively. Our approach in this work provides a feasible way for making 2D perovskite platelets for wide optoelectronic applications.

## 1. Introduction

Organic-inorganic halide perovskite are materials described by AMX_3_ formula, in which A is organic cation, M is metal cation and X is halogen anion [1]. Although perovskites have been discovered for more than one century, the use of perovskites in solar cells only happened in recent years [2,3,4,5,6,7,8,9,10]. Among the variety kinds of perovskites, methylammonium lead iodide perovskite (CH_3_NH_3_PbI_3_) has attracted intensive interest due to its extraordinary optoelectronic properties such as long electron/hole diffusion lengths, high optical absorption coefficient, and optimal bandgap [11,12]. These advantages have generated widely growing interest for diverse applications such as photodetectors [13,14,15,16], light-emitting diodes (LEDs) [17,18,19,20,21], waveguides [22], field effect transistors (FET) [23], lasers [24,25], etc. These applications requires high performance devices which highly depends on the perovskite material properties. To date, there are several methods to prepare CH_3_NH_3_PbI_3_ perovskite. Directly spin-coating perovskite compound solution to prepare perovskite films is the simplest method, which is suitable for use in solar cells with perovskite as the light harvester [18,20]. However, the material properties obtained by this method still cannot fully display the nature of perovskite, due to its inhomogeneous, polycrystalline structure and large surface roughness. Homogeneous and high crystalline perovskite film can be obtained by thermal evaporation, but this method needs dedicated equipment and can easily cause lead poisoning as the lead iodide (PbI_2_) vapor cannot be avoided in the experiment process [8,26]. Chemical vapor deposition (CVD) is promising for the synthesis of high quality perovskite with well-defined structures and morphologies [27,28], especially for two-dimensional (2D) CH_3_NH_3_PbI_3_ perovskite platelets. This method involves two steps: firstly, PbI_2_ platelets were grown on mica substrate under 350–510 °C, and then the as-grown PbI_2_ platelets were converted to CH_3_NH_3_PbI_3_ perovskites through inserting the CH_3_NH_3_I molecules into the PbI_2_ platelets under 120 °C [29]. Although high quality perovskite can be obtained by this method, it would still induce lead poisoning owing to the PbI_2_ vapor produced during the first growth step. Moreover, the high temperature growth process requires substrates that have high melting point (>350 °C), which are not suitable for flexible substrates with low melting point. Therefore, the need for developing other alternative routes to produce high quality perovskite platelets without lead halide vapour during the process on diverse substrates, especially on flexible substrates remains a challenge.

Previously, we introduce a two-steps method to produce high quality 2D perovskite platelets on SiO_2_/Si substrate [13]. The highest growth temperature during the process was around 180 °C. In this manuscript, we carefully control the growth temperature to below 100 °C, therefore high quality 2D perovskite platelets can be obtained on random substrates, especially on transparent, flexible and lower melting point substrates. Our method is applicable to any substrate as long as its melting point is higher than 100 °C. Moreover, photodetectors based on the as-produced 2D perovskite platelets were fabricated. The devices exhibit excellent photoresponse performance, including a high photoresponsivity of 8.3 AW^−1^, a high on/off ratio of ~10^3^, and a small rise and decay time of 30 and 50 ms, respectively. Considering the feasibility of preparing 2D perovskite platelets with different thickness on diverse substrates, especially on transparent, flexible and lower melting point substrates, the results in this paper would greatly extend the device applications of 2D perovskite.

## 2. Materials and Methods

The 2D CH_3_NH_3_PbI_3_ platelets were prepared by two steps. Firstly, 0.2 mg PbI_2_ powder was dissolved in 1 mL distilled water and heated at 100 °C for 1 h to get oversaturated PbI_2_ solution. After this, the hot PbI_2_ solution (around 100 °C) was dropped on the aimed heating substrates, including SiO_2_/Si, Si, mica, glass and PDMS. During this process, the PbI_2_ oversaturated solution would nucleate and form the 2D PbI_2_ platelets on the corresponding substrates. Secondly, CH_3_NH_3_I powder was put into the center of the furnace and the above obtained 2D PbI_2_ platelets were placed 10–15 cm downstream from the CH_3_NH_3_I powder in a CVD system (Hefei, Anhui, China) to convert to CH_3_NH_3_PbI_3_ perovskite. After that, 500 sccm Ar was introduced into the CVD system for 30 min to clear the air in the quartz tube. The Ar flow rate was then kept at 30 sccm to maintain the system pressure to be lower than 1 Torr. Afterword, the furnace was heated to 100 °C under a heating rate of 2.5 °C/min and kept at 100 °C for 5–30 min. Finally, we stopped heating and opened the furnace quickly. During this process, the CH_3_NH_3_I molecules would insert into the 2D PbI_2_ platelets to convert them into 2D CH_3_NH_3_PbI_3_ perovskite platelets. Moreover, the heating rate should not be very quick as higher heating rate usually induces temperature overshoot (>100 °C), and there would be more CH_3_NH_3_I molecules inserted into the PbI_2_ platelets than as needed. 

The photodetectors were fabricated by picking up a single organic ribbon by a mechanical probe and placed over the 2D perovskite platelet as an organic ribbon mask. After this, 30 nm Au was deposited on the 2D perovskite platelet by thermal evaporation. Finally, the organic ribbon mask was peeled off by a mechanical probe to form the source and drain electrodes over the 2D perovskite platelet. The morphology and structure of the as-grown 2D perovskite platelets were characterized by field-emission scanning electron microscopy (FESEM, Model S-4800, Hitachi, Tokyo, Japan), optical microscopy (Olympus BX51, Shinjuku, Tokyo, Japan), atomic force microscopy (AFM, Bruker, Dimension Icon SPM, Billerica, MA, USA) in the tapping model and X-ray diffraction (XRD, Bruker D8 advanced diffractometer, Cu-Kα radiation (λ = 1.54050 Å), Billerica, Massachusetts, United States ) scanned from 10 to 60° with a step of 0.02°. Chemical composition and crystal orientation of the 2D perovskite platelets were analyzed by X-ray photoelectron spectroscopy (XPS) and transmission electron microscope (TEM, FEI, Hillsboro, OR, USA, Tecnai G2 F20). Photoluminescence (PL) spectrum and mapping measurements were performed using a confocal microscope system (WITec, Ulm, Germany, alpha 300R) with 532 nm wavelength laser to excite the samples. The photoresponse properties of the photodetectors were characterized using the probe station (Cascade, Kingsey Falls, QC, Canada, M150) and a semiconductor property analyzer (Keithley, Cleveland, OH, USA, 4200) under 405 nm laser excitation.

## 3. Results

**Figure 1:**
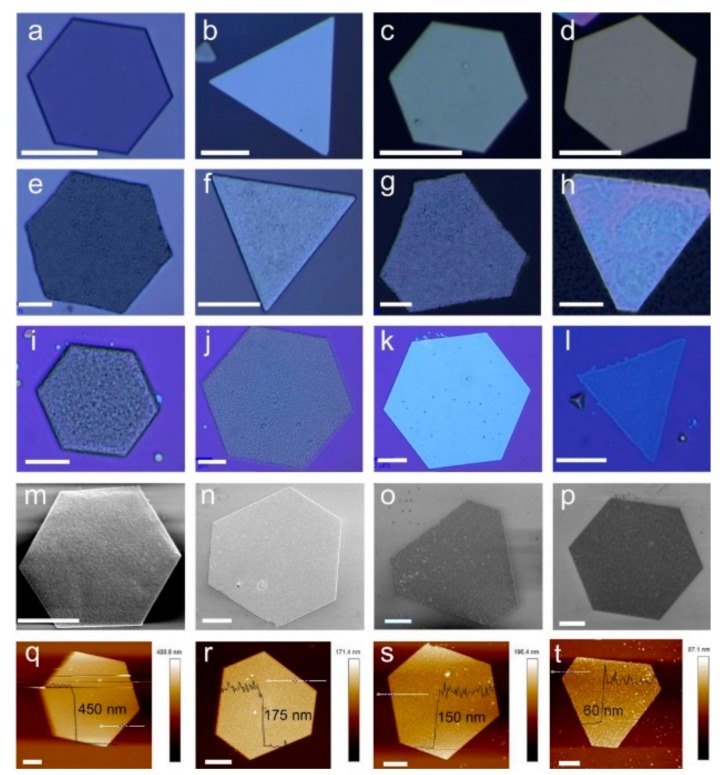
(**a**–**d**) Optical microscopy images of the hexagonal and triangular 2D PbI_2_ platelets on Si, mica, glass and PDMS substrates, respectively; (**e**–**h**) Optical microscopy images of the hexagonal and triangular 2D CH_3_NH_3_PbI_3_ perovskite on Si, mica, glass and PDMS substrates, respectively; (**i**–**l**) and (**m**–**p**) Optical microscopy and SEM images of 2D CH_3_NH_3_PbI_3_ perovskite with different thicknesses from hundreds of nanometers to single unit cell thick (2 nm) on SiO_2_/Si substrate; (**q**–**t**) AFM topography images of 2D CH_3_NH_3_PbI_3_ perovskite platelets with different thicknesses from 450 nm to 60 nm, respectively. All the scale bars are 10 μm.

Figure 1 shows the morphology of 2D PbI_2_ and CH_3_NH_3_PbI_3_ perovskite platelets characterized by optical microscopy, SEM and AFM. Figure 1a–d show the optical microscopy images of hexagonal and triangular 2D PbI_2_ on Si, mica, glass and flexible PDMS substrates, respectively. We can observe that the surfaces of all the 2D PbI_2_ platelets are smooth and uniform, no matter the substrate is rigid (Si, mica and glass)/flexible (PDMS) or smooth (Si and mica)/rough (PDMS and glass), which indicates that the toughness and roughness of the substrates do not play a key role during the PbI_2_ single crystal growth process. Figure 1e–h show the optical microscope images of 2D perovskite platelets on Si, mica, glass and PDMS substrates, respectively, which were converted from the single crystal PbI_2_ by inserting CH_3_NH_3_I molecules into them. After the conversion, the surfaces of perovskite platelets were found to be non-uniform and relatively rough compared to their counterparts before the conversion. The surface morphology change is induced by insertion of CH_3_NH_3_I molecules into the lattice of single crystal PbI_2_. Notably, we can obtain CH_3_NH_3_PbI_3_ perovskite on almost all kinds of substrates as long as its melting point is higher than 100 °C, which is the highest temperature during the whole process. Figure 1i–p show the optical and SEM images of perovskites with different thickness from hundreds of nanometers to a single-unit-cell thick (2 nm) on SiO_2_/Si substrate. Clearly, the surface of the 2D perovskites is more rough for the hundreds-nanometer-thick (Figure 1i) and the single-unit-cell-thick samples (Figure 1l), but the ten-nanometer-thick sample exhibits a much smoother surface (Figure 1k). AFM was used to explore the morphology and thickness of the 2D perovskite platelets, as shown in Figure 1q–t. Samples with thickness of 450 nm, 175 nm, 150 nm and 60 nm were measured. AFM morphology indicates a surface roughness of 24 nm, 22 nm, 18 nm and 13 nm, corresponding to 450 nm, 175 nm, 150 nm and 60 nm samples, respectively. From Figure 1s,t, we can even find small particles on the surface of thinner samples, this proves again that the CH_3_NH_3_I molecules insert into the PbI_2_ crystal and result in the surface morphology change.

**Figure 2:**
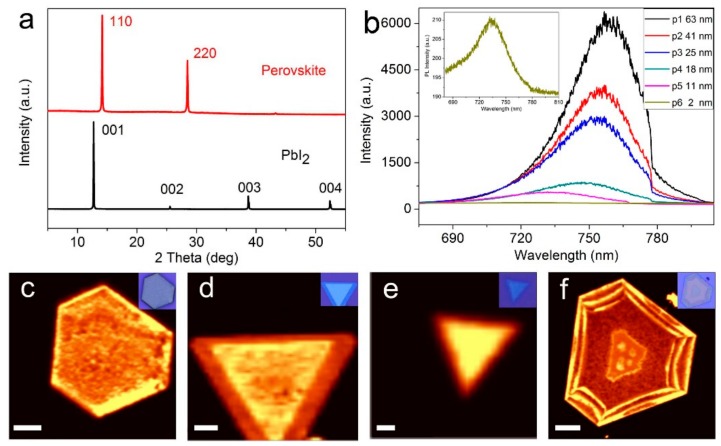
(**a**) XRD patterns of PbI_2_ platelets and corresponding converted 2D CH_3_NH_3_PbI_3_ platelets. The thickness of CH_3_NH_3_PbI_3_ platelets is 10 nm; (**b**) PL spectra of perovskite platelets with different thicknesses. The upper left inset shows the PL spectra of 2 nm perovskite; (**c**–**f**) PL mapping images of 2D CH_3_NH_3_PbI_3_ platelets with different thicknesses. As shown in (**c**,**d**), when the platelet is thicker than 10 nm, the PL intensities of the central part are lower than that of the boundary part. The upright insets in (**c**–**f**) show the corresponding optical microscopy images of 2D CH_3_NH_3_PbI_3_ platelets. The scale bars are 4, 7, 1 and 10 μm, respectively.

Figure 2a shows the XRD patterns of the 2D PbI_2_ and the corresponding CH_3_NH_3_PbI_3_ perovskite platelets on glass substrates with a thickness of 10 nm. The strong (110) and (220) diffraction peaks with 2θ located at 13.9° and 28.17° indicate that the obtained CH_3_NH_3_PbI_3_ perovskite is of tetragonal crystalline structure. Moreover, comparing the diffraction peaks of PbI_2_ and CH_3_NH_3_PbI_3_ perovskite, we can observe that the (001), (002), (003), (004) diffraction peaks of PbI_2_ disappeared after the conversion process, indicating that the PbI_2_ was completely converted to CH_3_NH_3_PbI_3_ perovskite crystals. Note that the normally observed (112), (211), (310) and (224) diffraction peaks in perovskite synthesized via solution method are not observed in our samples, attesting the fine crystal orientation of the converted CH_3_NH_3_PbI_3_ perovskite [30]. In order to characterize the optical properties of the converted perovskite, PL spectra were collected under 532 nm laser excitation at room temperature, as shown in Figure 2b and Figure S1a,b. Samples with different thickness that varies from 63 nm to single unit cell thick (2 nm) were marked by p1 to p6. The PL peak shifts towards shorter wavelength from 770 to 720 nm as the thickness of the perovskite decreases from 100 nm to single unit cell (2 nm), which is consistent with the previous report [13] and can be ascribed to the lattice expansion, namely, the structure relaxation of the in-plane crystal lattice could increase the optical band gap [31]. Moreover, we found that the PL intensity reduces dramatically by over 30 times as the film thickness decreases from 63 nm (marked by p1 in Figure 2b and Figure S1a,b) to single unit cell (2 nm, marked by p6 as shown in the upper left inset in Figure 2b and Figure S1a,b), owing to more excited charge carriers in the thicker perovskite under the laser irradiation. While for thinner perovskite, the carrier density is much less than that of thicker one, resulting in lower PL quantum yield efficiency. To further elucidate the relationship between the perovskite thickness and the corresponding PL intensity and peak position, PL mapping measurements were performed on 2D perovskite platelets, as shown in Figure 2c–f. When the perovskite thickness is larger than 100 nm, the boundary PL intensity is much higher than that in the central part (Figure 2c). In addition, this phenomenon still can be observed when the thickness decreases to 20 nm (inner triangular perovskite sheet in Figure 2d), although the intensity contrast is lower than that of the thicker ones. However, as the thickness decreases to thinner than 10 nm, the PL intensity is uniform over the whole crystal and no clear intensity contrast can be observed (inner triangular perovskite sheet in Figure 2e). While converting PbI_2_ to perovskite by inserting CH_3_NH_3_I molecules into it, the crystal boundary region of PbI_2_ is much easier to react with CH_3_NH_3_I molecules due to the large exposed edges than the central part during the conversion process, whereas, for the central part, the reaction first occurs at the surface and then towards inside the material. For thicker PbI_2_, the CH_3_NH_3_I molecules cannot insert into it through the surface easily, and consequently there are not enough CH_3_NH_3_I molecules involved into the reaction, resulting in the lower PL intensity from the central part than that from the boundary part. As the PbI_2_ thickness decreases, the insertion of CH_3_NH_3_I molecules becomes much easier though its surface, and thus the PL intensity difference between the central part and the boundary part is gradually reduced. As the thickness further decreases to 10 nm, sufficient number of CH_3_NH_3_I molecules can insert into PbI_2_ though its surface, thereby there is no obvious intensity difference between the central part and the boundary part (Figure 2e). Figure 2f proved again that the boundary PL intensity is much higher than the central part and the thicker perovskite have higher PL intensity as clarified in Figure 2b. To more intuitively demonstrate the above effect, PbI_2_ platelet is converted to CH_3_NH_3_PbI_3_ perovskite without supplying sufficient CH_3_NH_3_I molecules, and the resultant PL spectrum of the CH_3_NH_3_PbI_3_ perovskite is shown in Figure S2a,b, from which we can observe that the boundary exhibits higher PL intensity, and the intensity in the central part is very low, attesting the above analysis. 

**Figure 3:**
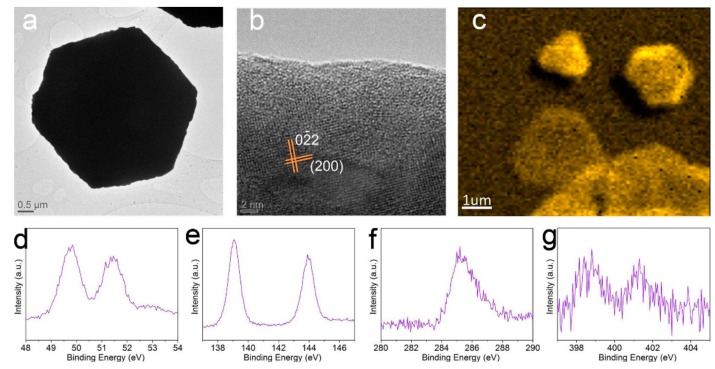
(**a**) TEM image of a 2D CH_3_NH_3_PbI_3_ platelet; (**b**) High-resolution TEM image of the 2D CH_3_NH_3_PbI_3_ platelet; (**c**) XPS mapping images of Pb element in 2D CH_3_NH_3_PbI_3_ platelets acquired with SPEM; (**d**–**g**) XPS spectra of Pb, I, C and N elements in 2D CH_3_NH_3_PbI_3_ platelets, respectively.

Figure 3a displays the TEM image of a hexagonal 2D perovskite platelet with a thickness of 10 nm. The corresponding high resolution TEM (HRTEM) image in Figure 3b shows clear lattice fringes with (200) and ($0\bar{2}2$) planes, further revealing the single crystalline structure of the converted perovskite platelet [13]. In order to understand the elemental arrangement of Pb in the perovskite platelets, scanning photoelectron microscopy (SPEM) was applied to obtain the XPS mapping image, as shown in Figure 3c. From the Pb mapping results, we can observe that the Pb arranged uniformly in the crystal after inserting the CH_3_NH_3_I molecules into the crystal. Moreover, single XPS spectra of Pb, I, C and N elements in the CH_3_NH_3_PbI_3_ perovskite were also acquired and shown in Figure 3d–g, respectively. The prominent C_1s_ peak located at 285.3 eV corresponds to the carbon atoms in CH_3_NH_3_PbI_3_ crystal, and a small amount of amorphous carbon located at 284.2 eV can also be seen, which may be due to the Si substrate contamination (Figure 3f). The I_4d_, Pb_4f_ and N_1s_ peaks are located at (49.8 eV, 51.4 eV), (138.8 eV, 144.3 eV) and (399 eV, 401.8 eV) respectively, which are in good agreement with previous reports [32,33,34].

## 4. Discussion

**Figure 4:**
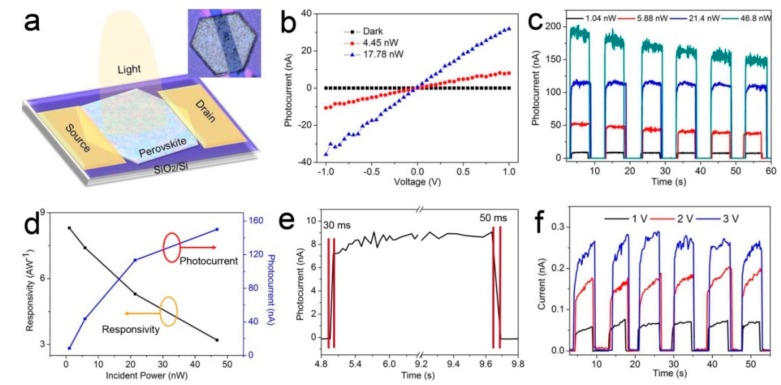
(**a**) Schematic of a 2D CH_3_NH_3_PbI_3_ platelet phototransistor. The upright inset shows the optical microscopy image of the 2D CH_3_NH_3_PbI_3_ platelet phototransistor; (**b**) *I*-*V* curves of the 2D perovskite-based device in dark and under light irradiation with different power; (**c**) Time-dependent photocurrent of the 2D CH_3_NH_3_PbI_3_ platelet with different incident power; (**d**) Dependence of photocurrent and photoresponsivity on incident light power; the blue and black dots correspond to original data; (**e**) Time photocurrent response excited at 405 nm laser. The rise time and the decay time are 30 ms and 50 ms, respectively; (**f**) Time dependent photocurrent of the device based on the 2D CH_3_NH_3_PbI_3_ platelet during the laser switching on/off process under positive source-drain voltage, V_sd_. V_sd_ is from 1 to 3 V.

Controlled growth of 2D perovskite platelets on different substrates enables us to probe their intrinsic optoelectrical properties. As an example, perovskite platelets were explored as the semiconducting channel of FETs on SiO_2_/Si substrate with two gold electrodes as source/drain electrodes and Si as the back gate. Schematic and optical microscopy image of a 2D CH_3_NH_3_PbI_3_ platelet phototransistor are shown in Figure 4a and Figure S3a. The results in Figure 4b display a nearly zero dark current and linear *I*-*V* curves under two different illumination powers with 405 nm laser excitation, indicating that the device has excellent photoresponse capability. The linear *I*-*V* curves indicate the Ohmic contact between perovskite and gold source/drain electrodes. Figure 4c shows the device response to pulsed light at different optical pumping power, from which we can observe that the device can be effectively switched “ON” and “OFF” while the laser source is turned on and off. The amplitude of the electrical signal is modulated by different light powers. Also from Figure 4c we can calculate the photocurrent to dark current ratio of our devices and the value can reach up to three orders of magnitude. Figure 4d shows the photoresponsivity and photocurrent as a function of the light power. It is found that both the photocurrent and photoresponsivity change nonlinearly with increasing the laser power. The photoresponsivity of our 2D perovskite based FET can reach up to 8.3 AW^−1^ under a bias voltage of 1 V, higher than the bulk perovskite film based devices (3 AW^−1^) but lower than perovskite crystal (40 AW^−1^) based ones with channel length reducing to 100 nm [15]. The response speed of our device is characterized by a rise time and a decay time of less than 30 and 50 ms, respectively, demonstrating a much faster response than the bulk perovskite film based devices. In addition, time-dependent photocurrents at different source-drain voltages are shown in Figure 4f and Figure S3b. The photocurrent can be significantly increased by increasing the source-drain voltage. As the laser is turned on and off, the photocurrent changes periodically, indicating a very good operation repeatability. The above results prove that our 2D perovskite platelets show excellent photoelectric properties and hold a potential for broader optoelectronics application, especially for applications that need low temperature processing.

## 5. Conclusions

In summary, high quality 2D CH_3_NH_3_PbI_3_ perovskite platelets were prepared by a two-steps method. By utilizing this method, we can produce 2D perovskites platelets with different thicknesses from hundreds of nanometers to single unit cell (2 nm) on different substrates, as long as the melting point of the substrate is higher than 100 °C. It was found that the PL characteristics of perovskite depends strongly on the platelet thickness, namely, thicker perovskite platelet has higher PL wavelength and stronger intensity, whereas, thinner perovskite exhibits opposite results. Moreover, photoelectric measurements confirm that our 2D perovskite platelets show excellent photoelectric properties. Phototransistors based on the 2D perovskite platelet exhibit a high photoresponsivity of 8.3 AW^−1^, a high on/off ratio of ~10^3^ with a small rise and a decay time of 30 and 50 ms, respectively. Considering the feasibility of preparing 2D perovskite platelets with different thickness on diverse substrates, especially on transparent, flexible and lower melting point substrates, our method would greatly extend the device applications of 2D perovskite.

## Supplementary Materials

The following are available online at http://www.mdpi.com/2079-4991/8/8/591/s1, Figure S1: Optical microscopy and PL mapping images of 2D CH_3_NH_3_PbI_3_ perovskite, Figure S2: Optical microscopy and PL mapping images of the converted 2D CH_3_NH_3_PbI_3_ perovskite without supplying sufficient CH_3_NH_3_I molecules during the conversion process, Figure S3: supplementary photoelectrical performance of the 2D CH_3_NH_3_PbI_3_ platelet phototransistor.
