# Supplementary material for: Controllable Synthesis of 2D Perovskite on Different Substrates and Its Application as Photodetector
Source: Nanomaterials (Basel). 2018 Aug 3;8(8):591. doi: 10.3390/nano8080591 (PMC6116234; doi:10.3390/nano8080591)
Supplement: Supplementary file 1 [file nanomaterials-08-00591-s001.pdf]

Controllable Synthesis of 2D Perovskite on Different Substrates and Its Application as Photodetector

Yunzhou Xue [1], Jian Yuan [2], Jingying Liu [3] and Shaojuan Li [2,*]

1 College of Chemistry and Environmental Engineering, Shenzhen University, Shenzhen 518000, China; xueyz@iccas.ac.cn

2 Collaborative Innovation Center of Suzhou Nano Science and Technology, Jiangsu Key Laboratory for Carbon-Based Functional Materials and Devices, Institute of Functional Nano and Soft Materials (FUNSOM), Soochow University, Suzhou 215123, China; jianyuan_cq@126.com

3 Department of Materials Science and Engineering, Monash University, Clayton, VIC 3800, Australia; jingying.liu@monash.edu

* Correspondence: sjli@suda.edu.cn; Tel.: +86-0512-6588-2337

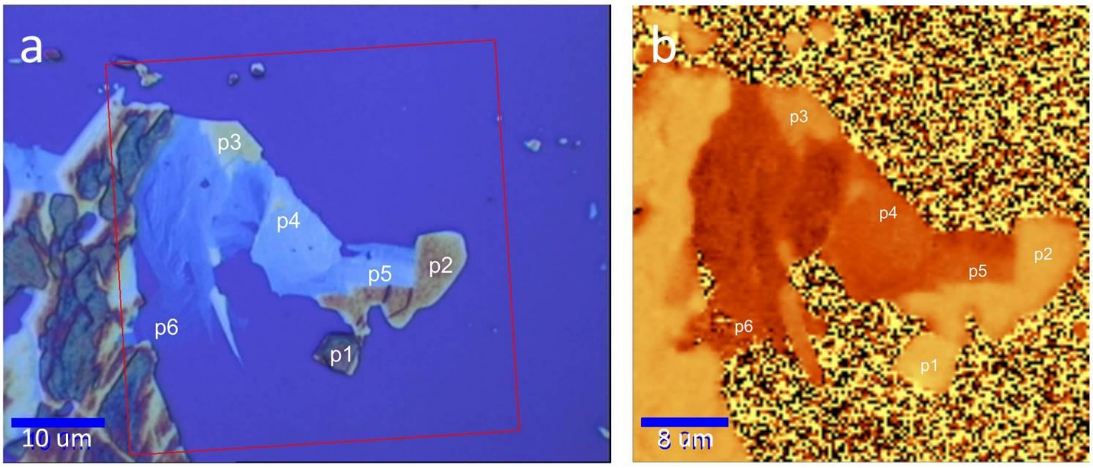

Figure S1. (**a**) $CH_3NH_3PbI_3$ perovskite with different thicknesses marked by p1 to p6. The thickness of P1 to P6 is measured to be 63 nm, 41 nm, 25 nm, 18 nm, 11 nm, 2 nm, respectively. (**b**) The corresponding PL mapping images of the $CH_3NH_3PbI_3$ perovskite acquired in the red square in (**a**).

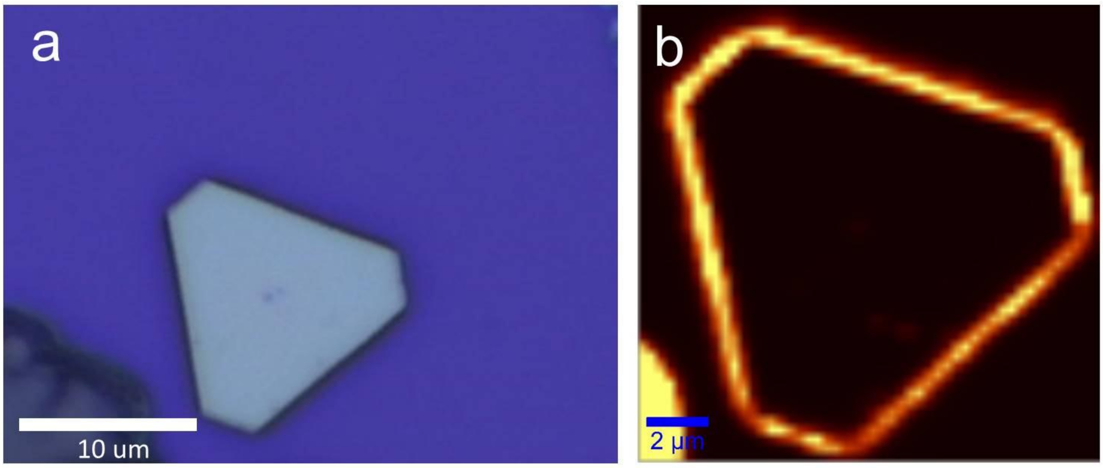

Figure S2. (**a**) Optical microscopy image of the converted $CH_3NH_3PbI_3$ perovskite without supplying sufficient CH_3NH_3I molecules during the conversion process on SiO_2/Si substrate. (**b**) PL mapping image of the converted $CH_3NH_3PbI_3$ perovskite in (**a**).

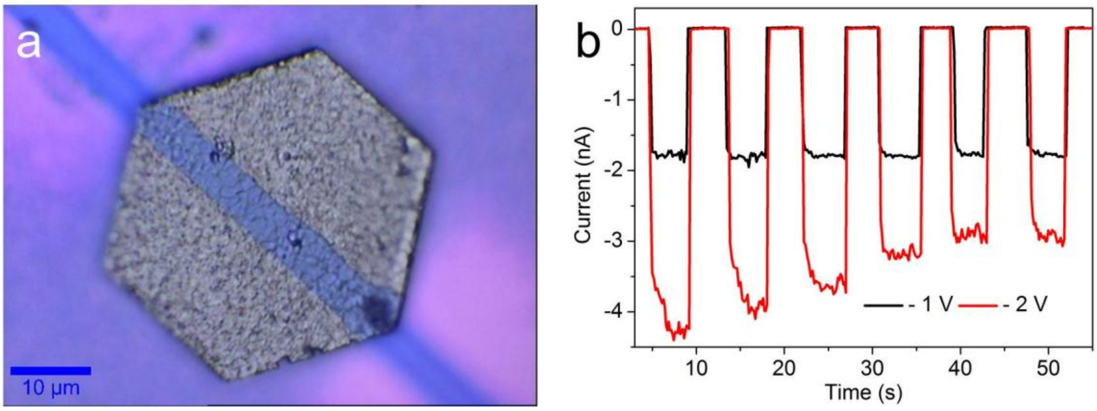

Figure S3. (**a**) Optical microscopy image of a 2D $CH_3NH_3PbI_3$ platelet phototransistor. (**b**) Time-dependent photocurrent of the 2D $CH_3NH_3PbI_3$ platelet phototransistor during the laser switching on/off process under negative source−drain voltages V_{sd} of −1 and −2 V.
